# Fabricating an Electrospray Ionization Chip Based on Induced Polarization and Liquid Splitting

**DOI:** 10.3390/mi12091034

**Published:** 2021-08-28

**Authors:** Lvhan Zhou, Qian Zhang, Xiangchun Xu, Xinming Huo, Qian Zhou, Xiaohao Wang, Quan Yu

**Affiliations:** 1 Open FIESTA, Tsinghua Shenzhen International Graduate School, Tsinghua University, Shenzhen 518055, China; zhoulh19@mails.tsinghua.edu.cn; 2 Division of Advanced Manufacturing, Tsinghua Shenzhen International Graduate School, Tsinghua University, Shenzhen 518055, China; qian.zhang.a@hotmail.com (Q.Z.); xuxiangchun1995@foxmail.com (X.X.); zhou.qian@sz.tsinghua.edu.cn (Q.Z.); 3 Division of Life Science & Health, Tsinghua Shenzhen International Graduate School, Tsinghua University, Shenzhen 518055, China; huoxinming15@163.com

**Keywords:** microfluidic chip, mass spectrometry, electrospray ionization, induced polarization

## Abstract

The coupling of the microfluidic chip to mass spectrometry (MS) has attracted considerable attention in the area of chemical and biological analysis. The most commonly used ionization technique in the chip–MS system is electrospray ionization (ESI). Traditional chip-based ESI devices mainly employ direct electrical contact between the electrode and the spray solvent. In this study, a microchip ESI source based on a novel polarization-splitting approach was developed. Specifically, the droplet in the microchannel is first polarized by the electric field and then split into two sub-droplets. In this process, the charge generated by polarization is retained in the liquid, resulting in the generation of two charged droplets with opposite polarities. Finally, when these charged droplets reach the emitter, the electrospray process is initiated and both positive and negative ions are formed from the same solution. Preliminary experimental results indicate that the coupling of this polarization-splitting ESI (PS-ESI) chip with a mass spectrometer enables conventional ESI-MS analysis of various analytes.

## 1. Introduction

A microfluidic chip, also known as a lab on a chip, is a biological, chemical, medical analysis platform for small amounts of fluids and microfluidics [1,2,3]. It can process sample preparation, reaction, and detection by using channels of tens to hundreds of micrometers in size [4]. Mass spectrometry (MS) can distinguish and identify chemical compounds by their mass-to-charge ratios. It has the characteristics of high sensitivity, fast response, and high precision, and miniaturization has become an important trend in its development [5,6,7,8,9,10]. The coupling of the microfluidic chip to mass spectrometry (Chip–MS) can greatly broaden the application fields of microfluidics technology and MS [11,12,13,14]. In Chip–MS analysis, many functions, such as separation, extraction, desalination, ionization, and so on, can be integrated on one microfluidic chip to simplify the experimental procedure, reduce the detection time, and facilitate the analysis of limited-quantity samples [15,16,17,18]. Moreover, a miniature mass spectrometer coupled with a microfluidic sample processing device is likely to become a powerful tool for field applications.

The key component to interface the microchip with a mass spectrometer is the ionization source, which is used to ionize the liquid samples for MS detection. So far, the two most commonly used ionization techniques in a Chip–MS system include matrix-assisted laser desorption/ionization (MALDI) and electrospray ionization (ESI) [19,20,21]. The former is suitable for off-line coupling between the microfluidic procedure and MS analysis, while the latter is much easier to perform and allows for continuous Chip–MS detection. Moreover, the flow rate used in the microfluidic chip is better matched with the ESI operation.

The generation of electrospray originated from the accumulation of charges in solution under the influence of a strong electric field [22]. Then, when some large solvent clusters are stripped from the solution, charged droplets are produced. The high voltage (HV) power source is an essential part of the ESI devices, but the method of applying the voltage is flexible. Traditionally, ESI can be achieved simply by applying an HV directly to the solution upstream of a spray tip. In addition, the use of auxiliary gas improves the nebulization and ionization efficiency, which is especially suitable for high flux ESI [23,24,25]. These approaches are also easy to implement on the chip, and hence, various ESI chips have been developed [26,27,28]. In these devices, the microfluidic channel replaces the capillary as the transfer path, and the chip edge serves as the spray emitter.

In traditional ESI, physical contact between the electrode and the solution may cause electrochemical modification of the analyte or sample contamination as well as dead volume [25,29,30]. To avoid these drawbacks, some ESI methods based on inductive effect have been proposed with no requirement of electrical contact [31,32,33,34]. So far, the induced ESI sources are mostly assembled by regular connectors and capillaries. In fact, the efficiency of inductive charging is lower than that of direct contact charging, so the induced ESI technique can only electrospray a small amount of solution. From this perspective, a microfluidic chip is another feasible way to perform induced electrospray.

In this study, we introduced a new approach to implement ESI on a chip based on electric field-induced polarization, which can avoid the contact between the electrodes and the solution. Through the specially designed microchannel and electrodes, a certain length of liquid column (or pulsed solution) can be polarized, split, and ionized. Both positive and negative ions can be produced simultaneously from the same liquid in the polarization-splitting ESI (PS-ESI) source. It is worth mentioning that the proposed PS-ESI approach is difficult to be implemented with conventional capillary and mechanical structures, which cannot construct smooth “Y” or “T” flow channels with low dead volume and continuously polarize the droplets during splitting. In contrast, as a microfluidic chip can build fine channels and electrodes and enables the precise control of the micro solution, it provides a suitable platform to verify the feasibility of the PS-ESI approach.

## 2. Materials and Methods

### 2.1. Materials and Instruments

Methanol and ethanol were purchased from ANPEL (Shanghai, China). We used dioxoprozine hydrochloride (DPZ) from 3Achemicals (Shanghai, China), chloramphenicol from Dr. Ehrenstorfer GmbH (Germany), and angiotensin (Ang I) from Aladdin (Shanghai, China) for verifying the devices. All of the above chemicals were analytical reagent grade. Polydimethylsiloxane (PDMS) elastomer base and a curing agent (Sylgard 184) were obtained from Dow Corning (Midland, MI, USA).

The oxygen plasma cleaner (PDC-M, Chengdu Mingheng Science & Technology Co., Ltd., Chengdu, China) was used for surface treatment and modification. The solution was injected using a syringe pump (KD Scientific, Holliston, MA, USA) and a microsyringe (Hamilton, Bonaduz, Switzerland). The channels and droplets on the microfluidic chips were observed by a microscope (IX71, OLYMPUS, Tokyo, Japan). The ion current was detected by a Keithley 6430 ampere meter. The developed ESI source was tested and characterized using an LCQ Fleet mass spectrometer (Thermo Fisher Scientific, Waltham, MA, USA).

### 2.2. Device Structure and Fabrication

**Figure 1:**
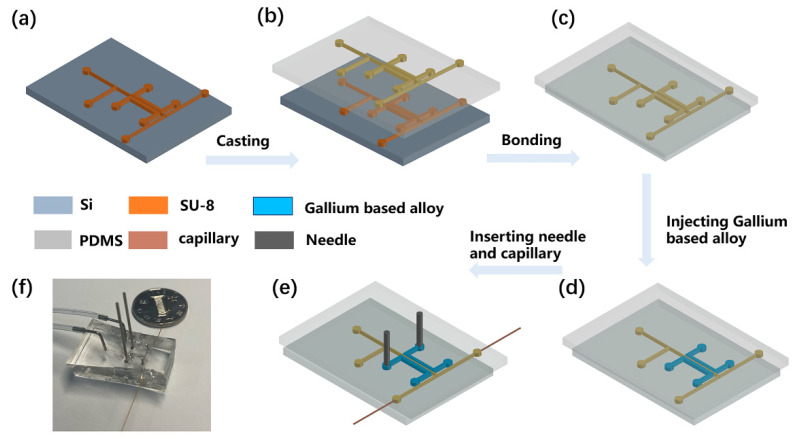
The flow chart of microfluidic chip fabrication. (**a**) Microfluidic channels on a silicon wafer. (**b**) Casting PDMS layer with microfluidic channel pattern from the silicon wafer. (**c**) Punching holes and bonding the PDMS layer. (**d**) Injecting Gallium based alloy into two paired-channels. (**e**) Inserting needles and fused-silica capillaries into PDMS. (**f**) A photograph of the 3D-electrode-integrated single-layer PDMS separator.

The PS-ESI chip was fabricated using soft lithography techniques. Microfluidic channel patterns of SU-8 2050 negative photoresist were produced by UV photolithography process on a silicon wafer with a thickness of 60 µm, with the following steps: soft baking, exposing photoresist, post-baking, developing photoresist layers, and hard baking. Then, the following procedures of fabricating microfluidic chips were performed using PDMS. Firstly, PDMS was fabricated by pouring a prepolymer of PDMS onto the mold (Figure 1a). A mixture of the prepolymer of PDMS elastomer and crosslinker with a weight ratio of 10:1 was poured onto the top and bottom of a SU-8 master mold. Then, the prepolymer was cured in an oven at 80 °C for 60 min and the PDMS layer was cast from the silicon wafer (Figure 1b). Holes were punched for inlets using metallic eyelets (tip diameter of 0.75 mm), and a PDMS layer was bonded to another PDMS substrate through the oxygen plasma (Figure 1c). After that, two stainless steel needles were inserted into the charging electrodes, which were filled with a conductive Gallium-based alloy, and two fused-silica capillaries (100 μm i.d., 375 μm o.d., 1.5 cm in length) were inserted into the outlets of the microfluidic chip channels to form the electrospray tip (Figure 1d,e). Finally, the PDMS chip was cured at 80 °C for 48 h to regain hydrophobicity, enhance bonding strength, and eliminate the MS background from the PDMS. The final microfluidic chip is shown in Figure 1f.

## 3. Results and Discussion

**Figure 2:**
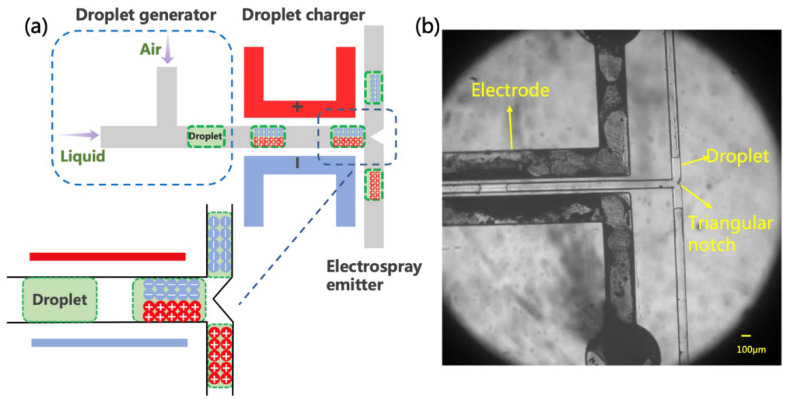
Schematic diagram and photograph of electrospray source. (**a**) The inset illustrates the presumption mechanism performed in the induction charge. Note that the scheme is not drawn to scale. (**b**) The droplet generation and movement under electrodes with voltage, and drops flow in both directions.

The formation of charged droplets is the first step in the ESI process. It can be implemented in many ways, but here, we propose a new method based on the polarization-splitting process of liquid using a unique microchannel structure. As shown in Figure 2a, the microfluidic charging chip is mainly composed of a droplet generator, an electrical polarization region, and a splitter. In the experiments, the injected sample solution was first cut into a short liquid column in the droplet generator. Air and spray solvent was injected from the two ends of the T-shaped channel, respectively. The width of the air inlet channel is 200 μm, and the width of the liquid inlet channel is 100 μm. Within this fluid-focus structure, air-in-water droplets were formed and then transferred to the polarization channel (100 μm width).

Using the fine microchannel configuration on the chip, we induced the polarization of a tiny amount of liquid flowing through the channel. In the polarization region, a pair of electrodes was located 50 μm away from the two sides of the main microchannel. When an appropriate potential is applied to the electrodes, an electric field is formed across the main channel to polarize the liquid solution. Namely, the positive and negative ions in the liquid tend to drift to the opposite sides of the solution surface along the electric field [33,35]. Similar observations have been described previously in an oil–water flow, in which the aqueous fluid was charged in an electric field [36,37]. Such a charge accumulation effect is affected by the electric field strength and the conductivity of the solution. Although this method was subsequently used for precise manipulation of microdroplets, the charging mechanism remains unclear, as the quantification of such a process has not been fully analyzed [38]. In any case, liquid polarization induced by the electric field creates the initial conditions for the generation of charged droplets.

In this paper, we propose a simple and unique method to produce charged droplets from the polarized solution by cutting the liquid column. As shown in Figure 2a, the main channel is divided into two branches (each with a width of 100 μm) after passing through the polarization zone. Different angles between the two branches, including 90 deg and 180 deg, were tentatively tried in the experiments. In particular, a triangular splitter was fabricated at the intersection in the 180 deg structure to better cut the liquid column from the middle. Then, the negatively and positively charged sub-droplets should be formed in the two channels, respectively. Similar splitting and charging effects were observed in these two chips. The angle was eventually set to 180 deg to minimize the mutual interaction between the two channels and facilitate the placement of the subsequent experimental setup. Figure 2b shows the process of droplet splitting, indicating that the liquid will flow smoothly and evenly to the two branches. During the splitting, the polarized charge is supposed to remain in the liquid. In other words, the originally neutral solution will split into two charged sub-droplets with opposite charges and then be transferred to the microchannel outlets.

**Figure 3:**
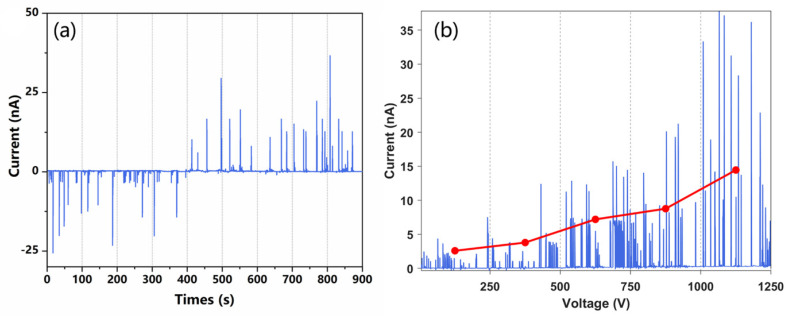
Current transients measured using counter electrodes. (**a**) A high potential of 1 kV was applied to the electrode to trigger the electrospray. Current detection at both outlets of the microfluidic chip. (**b**) Current transient measured at different spray voltages, the voltage is changed once every 100 s.

To verify the feasibility of the proposed charging approach, a Faraday plate connecting with a picoammeter is placed at the outlet of the channel to capture the charged liquid and detect the current. In the experiments, +1 kV and −1 kV voltages were applied to the two-paired electrodes, respectively, and the methanol aqueous solution was injected as the spray solvent. The acquired ion current of the liquid in one of the channel outlets is shown in Figure 3a. In the first 400 s, at the channel outlet near the positive electrode, a negative current was observed for each droplet. After 400 s, we switched the polarity of the voltage on the two electrodes, and then a positive current was detected on each polarized droplet at the same outlet. The results illustrated in Figure 3a show that charged droplets were formed in the chip, and their charge polarity was determined by the polarity of applied voltage.

In addition to voltage polarity, the effect of voltage intensity on the liquid charging was also investigated. A higher HV potential can increase the strength of the electric field, and then induce higher polarization and more charge accumulation in the solution. The solution was prepared at a 1:1 ratio (*v*/*v*) of methanol and ultrapure water with an addition of 0.1% formic acid to facilitate the formation of ions. As shown in Figure 3b, the intensity of the liquid current increased with the HV potential applied. It should be pointed out that the polarization-splitting method is only suitable for the charging of discontinuous and short liquid columns. If the polarized liquid is too long, the charge accumulation will be out of the polarization region, which will greatly reduce the efficiency of the charge separation during liquid splitting. In the experiments, the lengths of the initial droplets formed on the chip were not stable, which was probably due to the fluctuation of airflow. Furthermore, the induced polarization efficiency is lower than that of the electrical contact method, resulting in the fluctuation of the charge amount carried by each droplet. Nonetheless, the results illustrated in Figure 3 confirm that induced polarization combined with liquid cutting can indeed produce charged droplets from a neutral solution.

**Figure 4:**
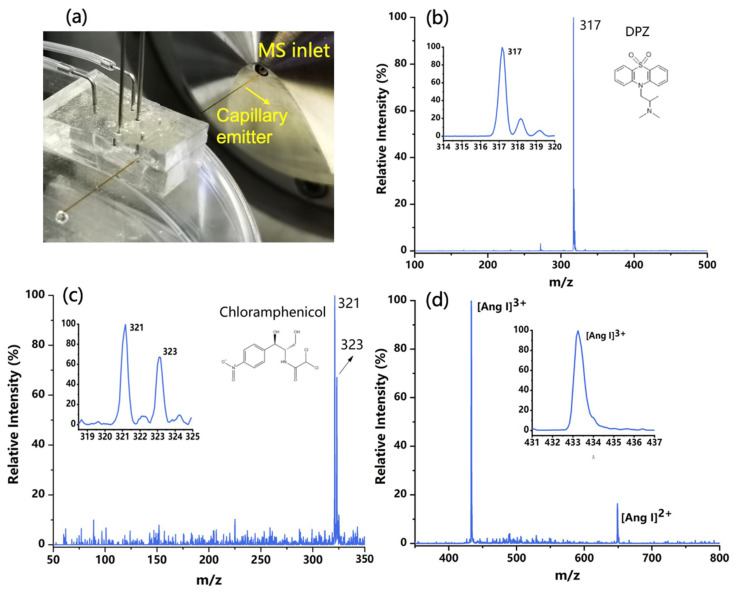
(**a**) The configuration of the chip ESI source coupling with mass spectrometry (MS). (**b**) Typical ESI mass spectrum of the DPZ solution in positive MS mode. (**c**) Typical ESI mass spectrum of the chloramphenicol in negative MS mode. (**d**) Typical ESI mass spectrum of the angiotensin (Ang Ⅰ) in positive MS mode.

The purpose of this study is to develop a new chip ESI source to promote the coupling of the microfluidic chip and mass spectrometry. After the formation of charged droplets, a spray tip is also needed to initiate an electrospray. Capillary tubing was then carefully inserted into the microchannel outlet to act as an emitter, which was placed several millimeters in front of the sampling interface of the mass spectrometer (see Figure 4a). The closer the capillary emitter is to the MS inlet, the stronger the ion signals are. Various sample solutions were injected into the chip to produce charged droplets and then transferred to the emitter. The liquid with sufficient charge density can initiate several steps, including desolvation, ion generation, and declustering [30], eventually becoming electrospray ions and being sampled into the mass spectrometer. The voltages applied to the two electrodes were +1 kV and −1 kV, which are lower than those used in conventional ESI sources. The relativity low voltage needed for spraying can be attributed to the small dimensions of the electrodes and microchannels fabricated in the microfluidic chip, as well as the tiny volume of the liquid polarized. In fact, this also presents an advantage of using microfluidic technologies to construct ESI sources.

The developed PS-ESI chip was applied for the analysis of various samples. In these experiments, the switching of the ionization mode was realized by either changing the voltage polarity or flipping the chip source. Some typical mass spectra are shown in Figure 4, including 50 ppm DPZ (*m*/*z* 317) collected in positive mode (in Figure 4b), 50 ppm chloramphenicol (*m*/*z* 321 and 323) acquired in negative mode (Figure 4c), and 100 ppm Ang Ⅰ in pure water obtained in positive mode with the charged ion peak signals of +2 and +3 appeared in the spectrum (Figure 4d). For each sample solution, the mass spectra acquired within 30 s were averaged. The ion peaks that appeared on the obtained spectra were consistent with those acquired from conventional ESI-MS detection.

**Figure 5:**
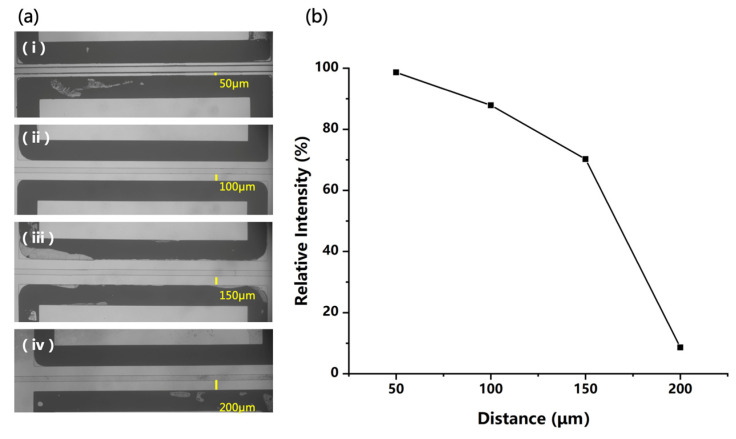
(**a**) Four different distances between microchannel and electrodes on microfluidics chips, and (**b**) the signal intensity of 50 ppm DPZ solution acquired with different distances.

When constructing an electric field in the chip for polarization of the liquid, parameters other than field strength also need to be considered. For example, a smaller distance between two electrodes can generate a stronger electric field but increase the probability of discharge. In this study, chips with different distances (50 μm, 100 μm, 150 μm, and 200 μm) between the microchannel and the electrodes were fabricated for structure optimization, as shown in Figure 5a. Then, a 50 ppm DPZ solution was analyzed in the positive mode using these chip sources by applying the same voltage on the electrodes. In this experiment, higher voltages of +2 kV and −2 kV were used to evaluate the possibility of discharge between electrodes. The plot of the ion intensity and the electrode distance is given in Figure 5b, which is a little different from what we expected. Reducing the electrode distance can provide a higher electric field for better polarization and ionization efficiency. However, when the distance was varied from 50 to 150 μm, although the calculated field strength decreased greatly, the ion signal acquired was only reduced by 30%. This may have been due to the complex process of charging the microdroplets with the polarization-splitting method, which would be affected by multiple factors and be difficult to quantify.

**Figure 6:**
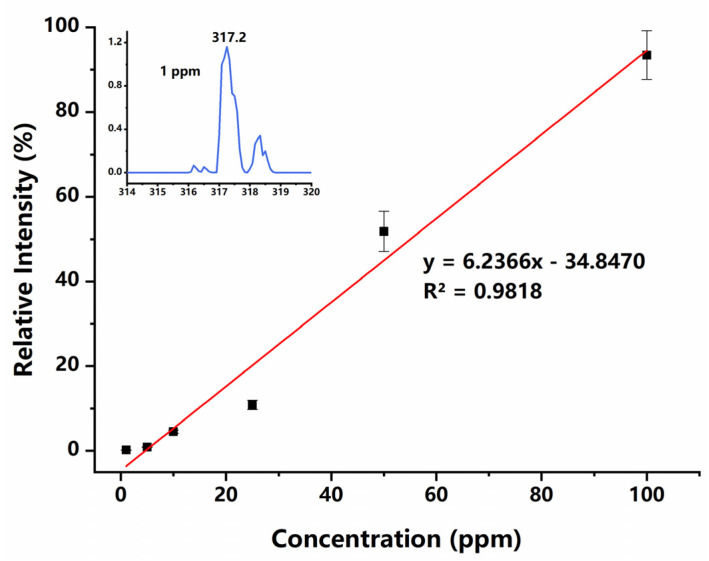
Calibration curve for DPZ measurements. Inset shows the acquired mass spectrum of 1 ppm DPZ solution.

To evaluate the quantitation ability of the PS-ESI source, a series of DPZ solutions with concentrations ranging from 1 ppm to 100 ppm were prepared and analyzed. The calibration curve obtained is shown in Figure 6, which indicates that the ion intensity has a moderately linear response with the concentration of DPZ. These experimental results prove that the PS-ESI chip can ionize the solution sample as the traditional ESI sources. Although the two channels of this chip can generate positive and negative sprays simultaneously, unfortunately, the two opposite polar sprays have not been detected at the same time in this experiment, because only one MS instrument was used with a switched detection mode for testing.

## 4. Conclusions

In the past decades, efforts have been made to facilitate the coupling of microfluidic chips with MS detection. One of the key technologies to achieve efficient chip–MS analysis is ionization. So far, the combination of microfluidic chips and MS analysis is mostly carried out through electrospray ionization. From another point of view, microfluidics offers us a new platform to perform ESI experiments that are difficult or impossible to perform using regular tubes, fittings, and devices. This research introduces a novel approach to generate ESI ions from the pulsed liquid on the chip. As indicated by the preliminary results, the proposed mechanism of generating ESI through the induced polarization and cutting of droplets has been proved to be feasible. Some features of this unique ionization strategy include no electrical contact, low DC voltage-driven, low sample consumption, and simultaneous generation of two polar sprays.
